# Development and Validation of Novel Prognostic Models for Immune-Related Genes in Osteosarcoma

**DOI:** 10.3389/fmolb.2022.828886

**Published:** 2022-04-06

**Authors:** Junqing Li, Li Su, Xing Xiao, Feiran Wu, Guijuan Du, Xinjun Guo, Fanguo Kong, Jie Yao, Huimin Zhu

**Affiliations:** ^1^ Minimally Invasive Spinal Surgery Center, Luoyang Orthopedic-Traumatological Hospital of Henan Province (Henan Provincial Orthopedic Hospital), Zhengzhou, China; ^2^ Scientific Research Center, Seventh Affiliated Hospital, Sun Yat-sen University, Shenzhen, China

**Keywords:** osteosarcoma, immune-related genes, tumor infiltrating immune cells, CD86, PGF

## Abstract

Immunotherapy has shown excellent therapeutic effects on various malignant tumors; however, to date, immunotherapy for osteosarcoma is still suboptimal. In this study, we performed comprehensive bioinformatic analysis of immune-related genes (IRGs) and tumor-infiltrating immune cells (TIICs). Datasets of differentially expressed IRGs were extracted from the GEO database (GSE16088). The functions and prognostic values of these differentially expressed IRGs were systematically investigated using a series of bioinformatics methods. In addition, CCK8 and plate clone formation assays were used to explore the effect of PGF on osteosarcoma cells, and twenty-nine differentially expressed IRGs were identified, of which 95 were upregulated and 34 were downregulated. Next, PPI was established for Identifying Hub genes and biology networks by Cytoscape. Six IRGs (APLNR, TPM2, PGF, CD86, PROCR, and SEMA4D) were used to develop an overall survival (OS) prediction model, and two IRGs (HLA-B and PGF) were used to develop a relapse-free survival (RFS) prediction model. Compared with the low-risk patients in the training cohort (GSE39058) and TARGET validation cohorts, high-risk patients had poorer OS and RFS. Using these identified IRGs, we used OS and RFS prediction nomograms to generate a clinical utility model. The risk scores of the two prediction models were associated with the infiltration proportions of some TIICs, and the activation of memory CD4 T-cells was associated with OS and RFS. CD86 was associated with CTLA4 and CD28 and influenced the infiltration of different TIICs. *In vitro* experiments showed that the knockdown of PGF inhibited the proliferation and viability of osteosarcoma cells. In conclusion, these findings help us better understand the prognostic roles of IRGs and TIICs in osteosarcoma, and CD86 and PGF may serve as specific immune targets.

## Introduction

Osteosarcoma is the most frequent primary solid malignant bone tumor with a high tendency for local invasion and early metastases, and it predominantly occurs in children and adolescents (Ritter and Bielack, 2010; Harrison et al., 2018). Since the 1980s, treatment has advanced from amputation to complex limb-sparing surgeries and incorporated multi-agent chemotherapy; beyond that, there has been no further progress (Harrison et al., 2018). The prognosis of patients with osteosarcoma remains suboptimal (Kager et al., 2017).

In recent years, important breakthroughs in immunotherapy have brought hope for the treatment of malignant tumors, including osteosarcoma (Rodig et al., 2018). Tumor-infiltrating immune cells (TIICs) play an important role in tumor progression and immunotherapy (Arneth, 2019; Yu et al., 2020; Chen et al., 2021). Tumor cells are lysed by IL-15-induced NK cells in patients (Buddingh et al., 2011). IL-12 can promote T-cell and B-cell proliferation, differentiation, and antibody formation and is used for tumor treatment (Chen et al., 2021). Regulatory T-cells (Tregs) orchestrate antitumor immunity by indirectly impeding T-cell activation *via* the CTLA-4-mediated inhibition of co-stimulatory signals of APCs (Tanaka and Sakaguchi, 2017). The inhibition of PD-1 or PD-L1 can reinvigorate the cytotoxic ability of T-cells and induce tumor regression (Iwai et al., 2005). As mentioned above, IL-15, IL-12, CTLA4, PD-1 and PD-L1 belong to immune-related molecules from ImmPort database (https://www.immport.org/resources) (Bhattacharya et al., 2018), and they have multiple effects on tumor development and immunotherapy (Arneth, 2019). Therefore, targeting and manipulating immune-related molecules can help control malignancies. As successful examples of immune-related molecules, anti-PD-1 and anti-PD-L1 antibodies have shown good clinical efficacy in treating some tumors, such as lung cancer and melanoma (Jiang et al., 2019). However, the results of current clinical trials suggest that most checkpoint inhibitors are less effective in treating solid tumors, including osteosarcoma (Ma et al., 2019; Chen et al., 2021). Therefore, it is necessary to explore specific immune-related targets in different tumors. Previous studies have indicated that immune-related genes (IRGs) can serve as effective prognostic biomarkers and potential targets of many tumors, such as lung adenocarcinoma (Zhang et al., 2019), ovarian cancer (Shen et al., 2019), and bladder cancer (Qiu et al., 2020). Therefore, an in-depth understanding of TIICs and IRGs will facilitate the identification of specific targeted molecules and provide new therapeutic directions for osteosarcoma.

In the present study, data on osteosarcoma were downloaded from TCGA, GEO, and TARGET. From these data, differentially expressed IRGs and hub genes were identified, and their potential functions and mechanisms were explored. An IRG-based prognostic model was developed and validated in this study. The association between the risk score and TIICs was also explored. Finally, our results showed that CD86 and PGF may serve as potential specific immune targets for osteosarcoma.

## Materials and Methods

### Data Processing

The data of 1793 IRGs (Supplementary Table S1) were extracted from the ImmPort database (https://www.immport.org/resources) (Chaussabel and Baldwin, 2014; Bhattacharya et al., 2018). IRGs were filtered out of gene expression profiles in the GSE16088 dataset by Perl script. Differentially expressed genes (DEGs) were identified from gene expression profiles of GSE16088 (Paoloni et al., 2009) obtained from osteosarcoma tissues and normal bone tissues using the “limma” package of R software (Ritchie et al., 2015). DEGs were identified based on adjusted *p*-values of <0.05 and log_2_|fold change| values of >1. Volcano plots were created using the “ggplot2” package (Maag, 2018), and heatmaps were created using the “pheatmap” package (Galili et al., 2018).

The training cohort underwent RNA sequencing, and clinical datasets were extracted from GSE39058 (Kelly et al., 2013). The validation cohorts were downloaded from TCGA-TARGET (https://portal.gdc.cancer.gov/) and TARGET (https://ocg.cancer.gov/). The cut-off value for the duration of survival was 10 years.

### GO Enrichment and KEGG Pathway Analysis

GO and KEGG pathway analyses were conducted using the R software“clusterProfiler” package (Yu et al., 2012). Functional enrichment analyses were performed for GO terms and KEGG pathways through a hypergeometric distribution with a significance threshold of *p* < 0.05. The enrichment results were visualized *via* the “ggplot2” packages.

### Identifying Hub Genes and Biology Network

The STRING database (https://string-db.org/) (Szklarczyk et al., 2019) was used to retrieve information about the protein-protein interactions (PPIs) of the differentially expressed IRGs. Cytoscape 3.7.2 software was used to construct the PPI network and visualize it. Critical modules and genes were selected from the PPI network using the MCODE plug-in. The degree cut-off was 2, the node score cut-off was 0.2, and the K-core was cut-off was 4. Two topological features from cytoHubba plug-in, degree and betweenness, were used to identify candidate hub genes. Nodes’ scores were calculated by cytoHubba plug-in. The genes with the top 20 highest nodes’ scores were considered as candidate hub genes. Finally, the hub genes were identified based on the overlap of the results of the degree topological method, betweenness topological method, and MCODE method.

### Construction and Validation of Prognostic Models

First, we collated and combined the mRNA expression data and clinical data from GSE39058. Subsequently, univariate Cox regression analysis of the DEGs was performed *via* the “survival” package, and statistical significance of the candidate genes was determined using a log-rank test. Subsequently, a multivariate Cox regression model was established *via* multivariate Cox regression analysis, and the risk score of each patient was calculated. The formula for the risk score was as follows: risk score = coef_gene1_ × Exp _gene1_ + coef_gene2_ × Exp_gene2_ + coef_genei_ × Exp_genei_. Patients were assigned to the low-risk or high-risk groups according to the median risk score. Overall survival (OS) and relapse-free survival (RFS) of patients in the two subgroups were compared using the log-rank test. The Kaplan-Meier survival ROC package was used to develop ROC curves to evaluate the predictive capability of the aforementioned prognostic model. In addition, the risk score for the validation cohort from the TARGET datasets was calculated using the established model of the training cohort. The survival and ROC curves of the validation cohort were visualized using the aforementioned methods. Ultimately, to predict OS and RFS more conveniently, we established a nomogram using the “rms” package.

### Tumor-Infiltrating Immune Cell Analysis Based on CIBERSORT

The tumor-infiltrating immune cells (TIICs) of the GSE16088 and TARGET datasets were assessed using the CIBERSORT analytical tool (https://cibersort.stanford.edu/) (Newman et al., 2015). The abundance ratio matrix of the 22 immune cells was obtained at *p* < 0.05.

### Cell Culture and siRNA Transfection

MG63 and U2OS cell lines were purchased from the Cell Bank of the Chinese Academy of Sciences (Shanghai, China). MG63 and U2OS cells were cultured in DMEM (Biological Industries, Shanghai, China) containing 10% fetal bovine serum (Biological Industries, Shanghai, China). The cells were grown at 37°C in an atmosphere of 5% CO_2_. GenOFF PGF siRNA and negative control siRNA oligonucleotides were designed and synthesized by RiboBio (Guangzhou, China). The sequences of si-1 and si-2 are shown in Supplementary Table S2. The siRNA transfections were performed using the Ribo FECT™CP Transduction Kit (Ribobio, Guangzhou, China) according to the manufacturer’s instructions.

### RNA Extraction and Quantitative Real-Time PCR

Following the manufacturer’s instructions, total RNA was retrieved with AG RNAex Pro Reagent (Accurate Biology, Changsha, China) and was reverse-transcribed into cDNA using the Evo M-MLV RT Premix Kit (Accurate Biology). Quantitative real-time PCR (RT-PCR) assays were performed using the SYBR^®^ Green Premix Pro Taq HS qPCR Kit (Accurate Biology) according to the manufacturer’s protocols. The primer sequences are listed in Supplementary Table S2.

### Western Blotting

Cells were lysed with RIPA lysis buffer (Beyotime Biotechnology, Shanghai, China) and separated by SDS-PAGE. The proteins were transferred to polyvinylidene fluoride membranes (Merck Millipore, Billerica, MA, United States). Subsequently, the PVDF membranes were soaked in 5% BSA solution for at least one hour at room temperature, followed by incubation with different primary antibodies at 4°C overnight. On the second day, the PVDF membranes were incubated with secondary antibodies for one hour at room temperature after washing three times. After washing with TBST solution, the PVDF membranes were visualized using a BeyoECL Plus Kit (Beyotime Biotechnology). Anti-β-actin (20536-1-AP) and anti-PGF antibody (10642-1-AP) were purchased from Proteintech (Wuhan, China).

### Cell Proliferation and Plate Clone Formation

Cell proliferation was detected using the Cell Counting Kit-8 (CCK8, Beyotime Biotechnology) according to the manufacturer’s instructions. The cells were cultured in a 96-well plate (2 × 10^3^ cells/well) at 37°C for 0, 24, 48, and 72 h. For the plate clone formation assay, 500 cells were seeded into 6-well plates and cultivated for 14 days. After removing the medium, the cells were washed with PBS, fixed with 4% paraformaldehyde for 20 min, and stained using 1% crystal violet for 20 min at room temperature. The number of colonies with >50 cells was counted under a light microscope, and the colonies were visualized.

### Statistical Analysis

R software was used for most of the bioinformatics and statistical analyses in this study, including RNA-seq data normalization and transformation, CIBERSORT, DEG analysis, survival analyses, ROC analysis, and Spearman rank correlation analysis. Univariate and multivariate Cox regression analyses were performed using the “coxph” command of the “survival” package.

For the *in vitro* experiments, all quantitative data are presented as mean ± standard deviation of three independent experiments. Differences between the three groups were analyzed with one-way ANOVA using GraphPad Prism 8.0 (GraphPad, La Jolla, CA, United States). Statistical significance was set at *p* < 0.05.

## Results

### Differentially Expressed IRGs in Osteosarcoma

**FIGURE 1:**
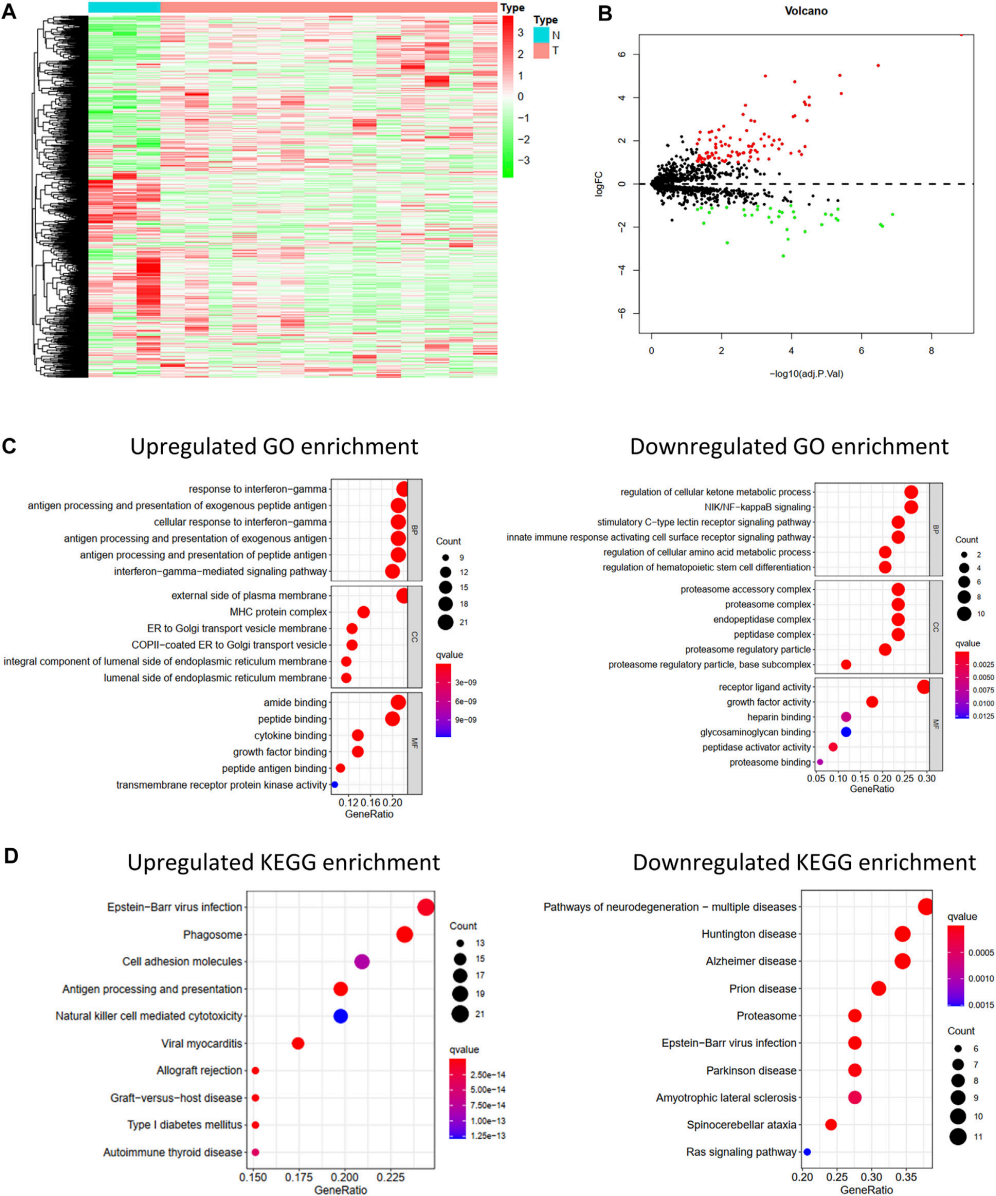
Differentially expressed IRGs and its function enrichment analysis. Heatmap**(A)** and volcano plot **(B)** of 1,793 immune-related genes in normal bone tissues and osteosarcoma tissues from GSE16088. **(C)** GO pathway enrichment of upregulated and downregulated IRGs. **(D)** KEGG pathway enrichment of upregulated and downregulated IRGs.

Differentially Expressed IRGs were identified using the “limma” package for R software from the GSE16088 data covering normal bone tissues and tumor tissues. The heat map of IRG expression in GSE16088 is shown in Figure 1A. Finally, 129 IRGs were differentially expressed; 95 were upregulated and 34 were downregulated (Figure 1B, Supplementary Table S3).

### GO and KEGG Pathway Enrichment Analysis

To explore the functions of these differentially expressed IRGs and their potential mechanisms in osteosarcoma, GO and KEGG functional analyses of these downregulated and upregulated IRGs were performed *via* the “clusterProfiler” package for R software.

As shown in Figure 1C, significant differences in the functional enrichment of the downregulated and upregulated IRGs were observed. For the cellular component (CC), the upregulated IRGs were enriched on the external side of the plasma membrane, MHC protein complex, ER to Golgi transport vesicle membrane, COPII-coated ER to Golgi transport vesicle, an integral component of the luminal side of the endoplasmic reticulum membrane, and the luminal side of the endoplasmic reticulum membrane. The downregulated IRGs were enriched in the proteasome accessory, proteasome, endopeptidase, and peptidase complexes, proteasome regulatory particle, and base subcomplex. Differences in the CC correspond to the different molecular functions. Regarding molecular function (MF), the upregulated IRGs play roles in amide binding, peptide binding, cytokine binding, growth factor binding, peptide antigen binding, and transmembrane receptor protein kinase activity, and the downregulated IRGs play roles in receptor-ligand activity, growth factor activity, peptidase activator activity, and proteasome binding. Biological process (BP) analysis showed that the upregulated IRGs participated in the response to interferon-gamma, antigen processing and presentation of exogenous and peptide antigens, and interferon-gamma-mediated signaling pathways, and the downregulated IRGs participated in the regulation of the cellular ketone metabolic process, NIK/NF-kappaB signaling, stimulatory C-type lectin receptor signaling pathway, and innate immune response-activating cell surface receptor signaling pathway.

Moreover, the KEGG pathway enrichment analysis showed differences between the upregulated and downregulated IRGs (Figure 1D). The upregulated IRGs were enriched in phagosomes, cell adhesion molecules, antigen processing and presentation, and natural killer cell-mediated cytotoxicity, and the downregulated IRGs were enriched in the proteasome and Ras signaling pathways.

### Identify Hub Genes and Biology Network

**FIGURE 2:**
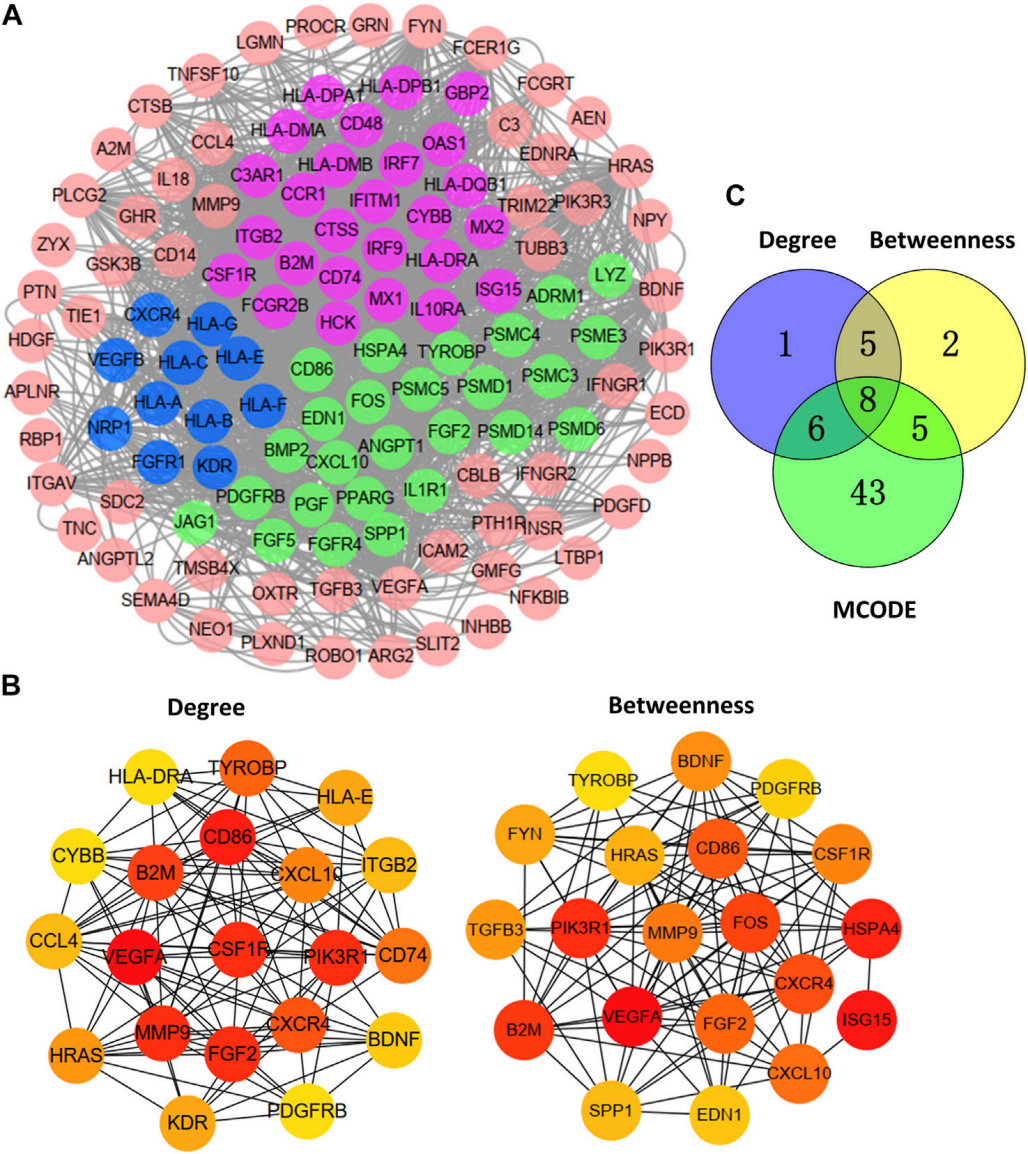
Hub genes and biology network. **(A)** Protein-protein interaction (PPI) network of 129 differentially expressed IRGs and three critical modules. **(B)** Identifying the first 20 IRGs and constructing the corresponding PPI network using the degree and betweenness topological method. **(C)** Venn calculation applied to identify eight hub IRGs.

The PPI network of 129 DEGs was mapped by STRING and reconstructed using the Cytoscape MCODE plug-in (Figure 2A). Three critical modules were screened (Figure 2A). We selected the first 20 genes and constructed the corresponding PPI network using the degree and betweenness topological methods (Figure 2B). By crossing the results of the three methods (Figure 2C), eight genes were identified as hub genes: CXCR4, PDGFRB, CXCL10, B2M, CD86, CSF1R, TYROBP, and FGF2.

### Construction and Validation of Prognostic Model

**FIGURE 3:**
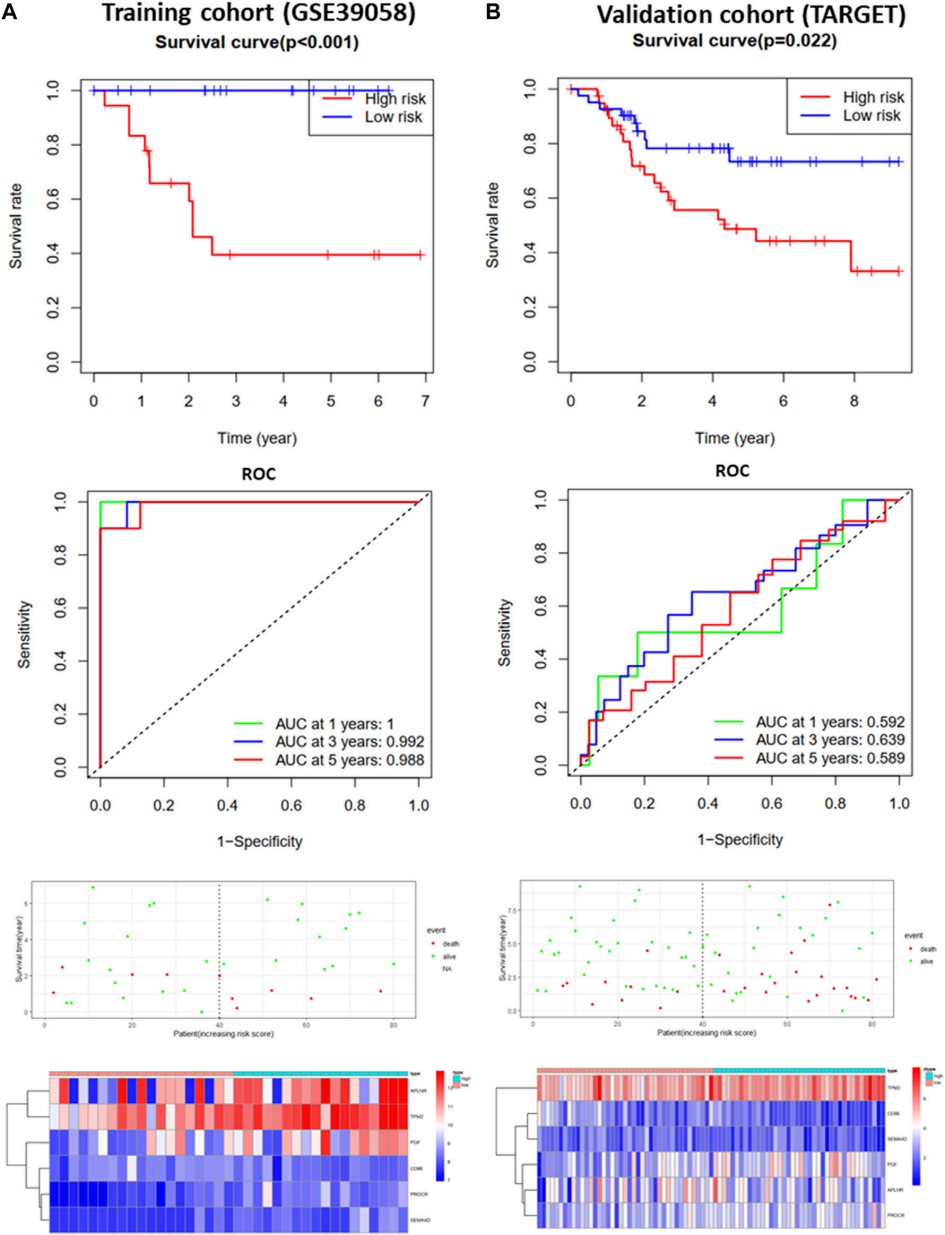
Development and validation of the OS-prediction model for osteosarcoma. **(A)** Survival curve, ROC curve, survival status, and heat map for low- and high-risk subgroups in training cohort (GSE39058). **(B)** Survival curve, ROC curve, survival status, and heat map for low- and high-risk subgroups in validation cohort (TARGET).

**FIGURE 4:**
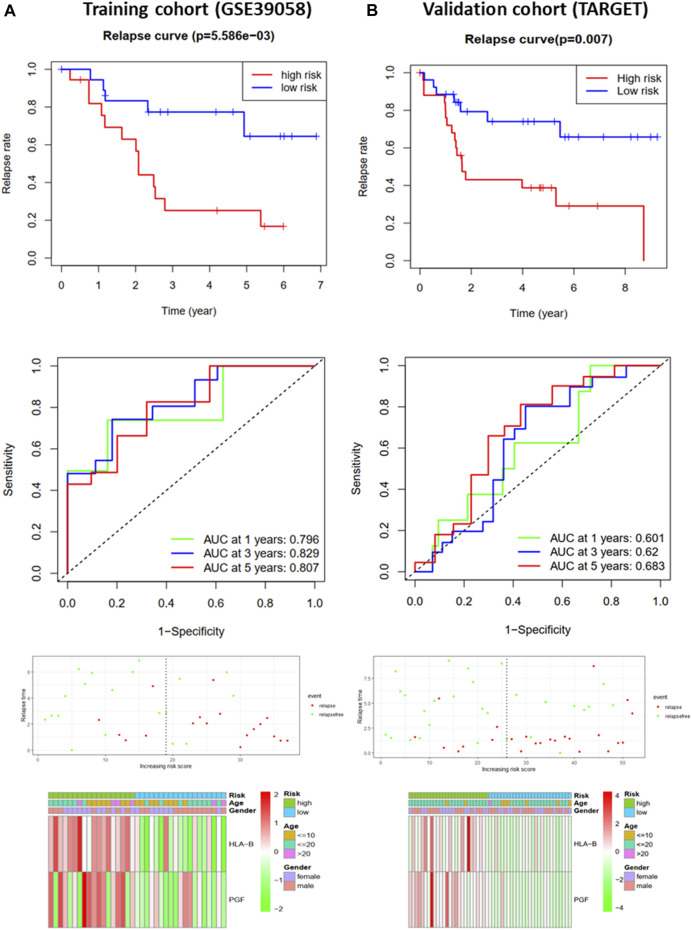
Development and validation of the RFS-prediction model for osteosarcoma. **(A)** Relapse curve, ROC curve, relapse status, and heat map for low- and high-risk subgroups in training cohort (GSE39058). **(B)** Relapse curve, ROC curve, relapse status, and heat map for low- and high-risk subgroups in validation cohort (TARGET).

To further analyze the effects of IRGs on the prognosis of osteosarcoma patients, we first performed univariate Cox regression analysis to assess the impact of DEGs on OS and RFS in GSE39058 sets. The results suggested that nine and eight candidate IRGs were significantly associated with OS and RFS, respectively (Supplementary Image S1). Subsequently, the impact of these candidate IRGs on OS or RFS was established using multivariate Cox regression analysis. Finally, APLNR, TPM2, PGF, CD86, PROCR, and SEMA4D were selected as features of the OS prediction model as follows: risk score = (−4.377*Exp APLNR) + (12.781*Exp TPM2) + (5.755*Exp PGF) + (−11.809*Exp CD86) + (11.874*Exp PROCR) + (−3.496*Exp SEMA4D). HLA-B and PGF were selected as features for the RFS prediction model, and the formula was as follows: risk score = (0.623 * Exp HLA-B) + (0.568 * Exp PGF). The risk score for each patient was assessed. Based on the median risk score, patients from the training cohort (GSE39058) were assigned to the low-risk and high-risk groups. The results of the survival analysis demonstrated that patients in the high-risk group had significantly poorer OS (*p* < 0.001, Figure 3A) and RFS (*p* < 0.01, Figure 4A) than those in the low-risk group. To further evaluate the prognostic utility of these IRGs, we subsequently performed a time-dependent ROC analysis, and the results revealed that the areas under the ROC curve (AUCs) were 1, 0.991, and 0.988 (Figure 3A) for the OS model and 0.796, 0.829, and 0.807 for the RFS model for 1, 3, and 5 years (Figure 4A). The survival status of the patients and the heat maps for the expressions of these are shown in Figures 3A, 4A.

The OS and RFS models were applied to the validation cohort from the TARGET sets to validate them. For the OS model, the patients with high risk scores had significantly worse OS than those with low risk scores in the validation cohort (*p* < 0.05, Figure 3B). The AUCs for OS in the validation cohort were 0.592, 0.639, and 0.589 for 1, 3, and 5 years (Figure 3B). The survival status of patients and the heat map for the expressions of these IRGs in the validation cohort are shown in Figure 3B. For the RFS model, patients with high risk scores had worse RFS than those with low risk scores in the validation cohort (*p* < 0.01, Figure 4B). The AUCs for the RFS in the validation cohort were 0.601, 0.62, and 0.683 for 1, 3, and 5 years (Figure 4B). The survival status of patients and the expression heat maps for the expressions of these IRG in the validation cohort are shown in Figure 4B. In summary, seven prognosis-related IRGs were identified, and the OS and RFS models were reliable in predicting the outcomes of patients with osteosarcoma.

### Building a Predictive Nomogram

**FIGURE 5:**
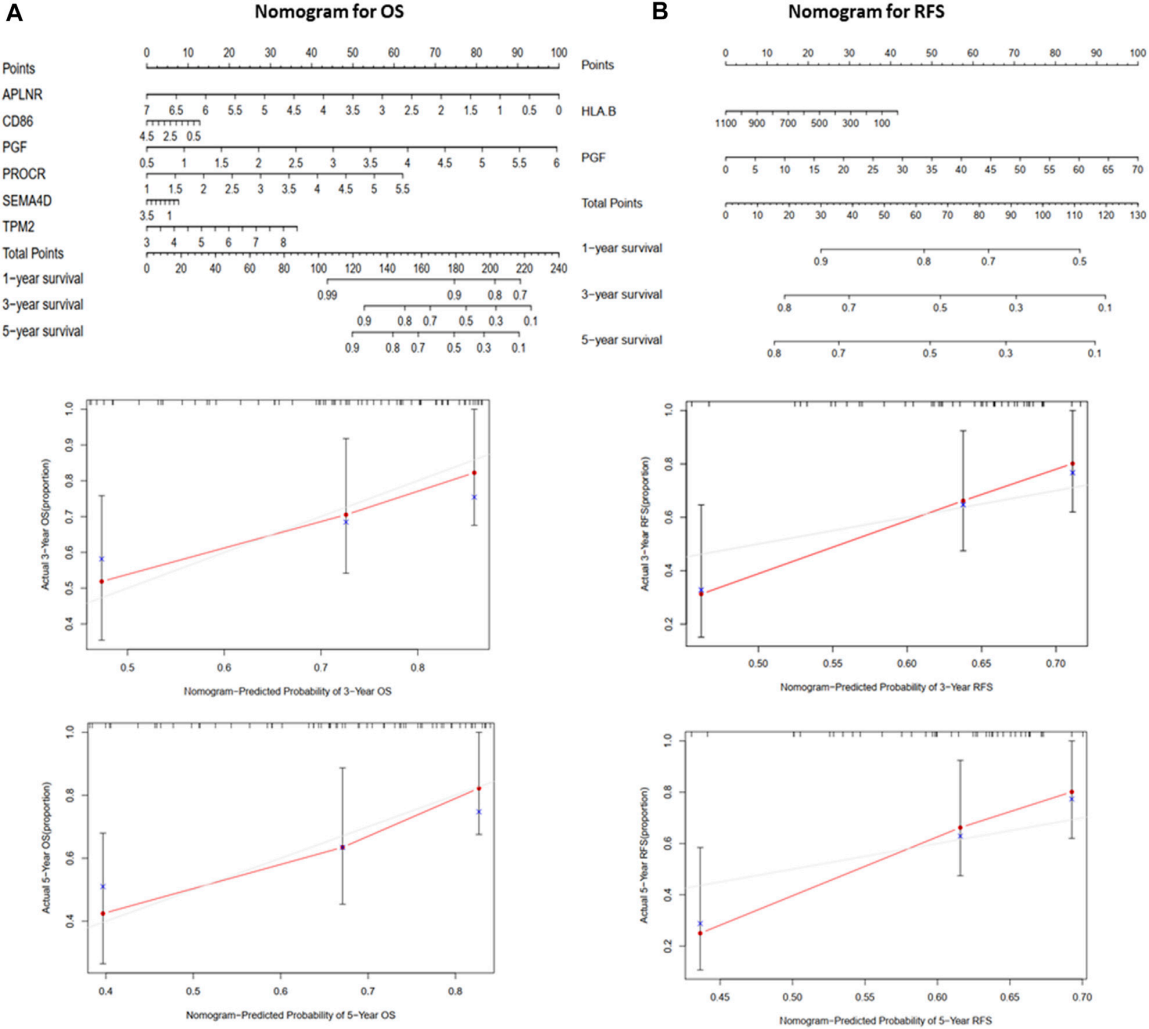
The nomograms for predicting 1-, 3-, and 5-year OS and RFS of the validation cohort (TARGET). **(A)** Nomogram for predicting 3- and 5-year OS and calibration plots. **(B)** Nomogram for predicting 3- and 5-year RFS and calibration plots.

A nomogram (Figure 5) was constructed to generate a clinically practical model that would enable physicians to predict the OS and RFS of osteosarcoma patients using prognosis-related IRGs. Based on the results of multivariate Cox analysis of the validation cohort, each variable was assigned a corresponding point based on the point scale obtained using this nomogram. A horizontal line was drawn to determine the points of each variable. The patient’s total score was calculated by adding up the points of all the variables, based on which the 1-, 3-, and 5-year survival rates were estimated.

### Tumor-Infiltrating Immune Cells Based on CIBERSORT

**FIGURE 6:**
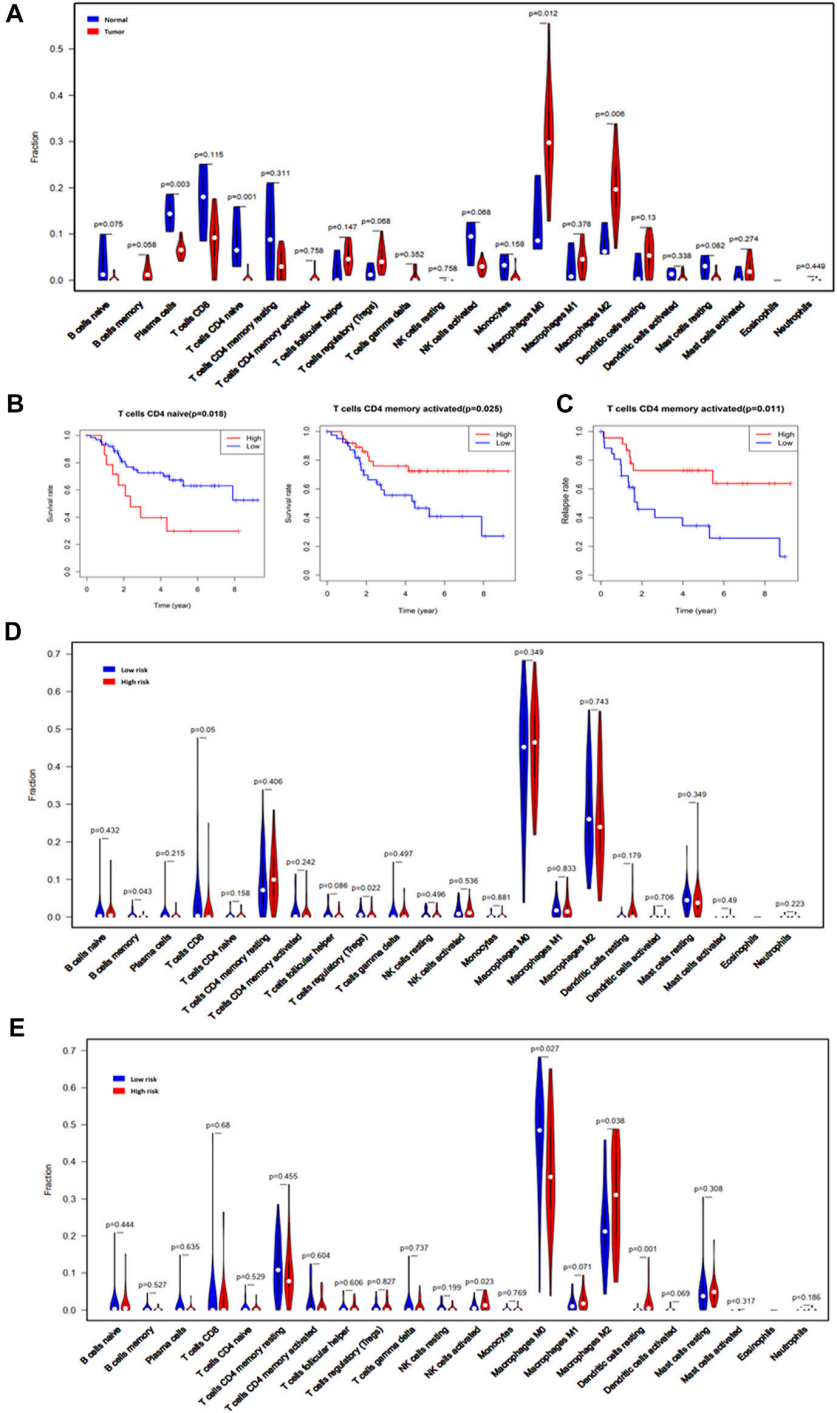
Tumor-infiltrating immune cells based on CIBERSORT. **(A)** Violin plot comparing the proportions of TIICs in normal bone tissues and osteosarcoma tissues in GSE16088. **(B)** The OS analysis of naïve and memory-activated CD4 T-cells for the validation cohort (TARGET). **(C)** The RFS analysis of the memory CD4 T-cells for the validation cohort (TARGET). **(D)** Violin plot comparing the proportions of TIICs associated with low and high risk scores designated by the OS prediction model for the validation cohort (TARGET). **(E)** Violin plot comparing the proportions of TIICs associated with the low and high risk scores designated by the RFS prediction model for the validation cohort (TARGET).

To explore the impact of the risk score on TIICs, the infiltration proportions of 22 immune cells in the GSE16088 and TARGET sets were calculated using the CIBERSOT algorithm. For the osteosarcoma samples, M0 and M2 macrophages were the major constituents of TIICs (Supplementary Image S2). Compared with normal bone tissues, the infiltration proportions of plasma cells (*p* = 0.003) and naïve CD4 T-cells (*p* = 0.001) were significantly reduced in osteosarcoma tissues, while the infiltration proportions of M0 macrophages (*p* = 0.012) and M2 macrophages (*p* = 0.006) were increased (Figure 6A). We analyzed the relationships between the TIICs and OS or RFS. The results suggested that patients with high infiltration proportions of naïve CD4 T-cells had poorer OS than those with low infiltration proportions (*p* = 0.018, Figure 6B), while patients with high proportions of activated memory CD4 T-cells had longer OS (*p* = 0.025, Figure 6B) and RFS (*p* = 0.011, Figure 6C) than those with low infiltration proportions.

Compared with the low-risk group designated by the OS model, the proportions of B-cell memory (*p* = 0.043, Figure 6D) and TRegs (*p* = 0.022, Figure 6D) were significantly reduced in the high-risk group. The infiltration proportions of the activated NK cells (*p* = 0.023), M2 macrophages (*p* = 0.038), and resting dendritic cells (*p* = 0.001) in the high-risk group designated by the RFS model were significantly higher than those in the low-risk group, while the infiltration proportions of M0 macrophages in the high-risk group designated by the RFS model were significantly lower than those in the low-risk group (Figure 6E). In addition, the associations between the seven prognosis-related IRGs and TIICs were analyzed, and these IRGs had different associations with different TIICs (Supplementary Image S3). All these results suggest that the seven prognosis-related IRGs interact with some TIICs, and they all influence the progression and prognosis of osteosarcoma. Key links still need to be further explored.

### Potential Targets of Osteosarcoma Immunotherapy

**FIGURE 7:**
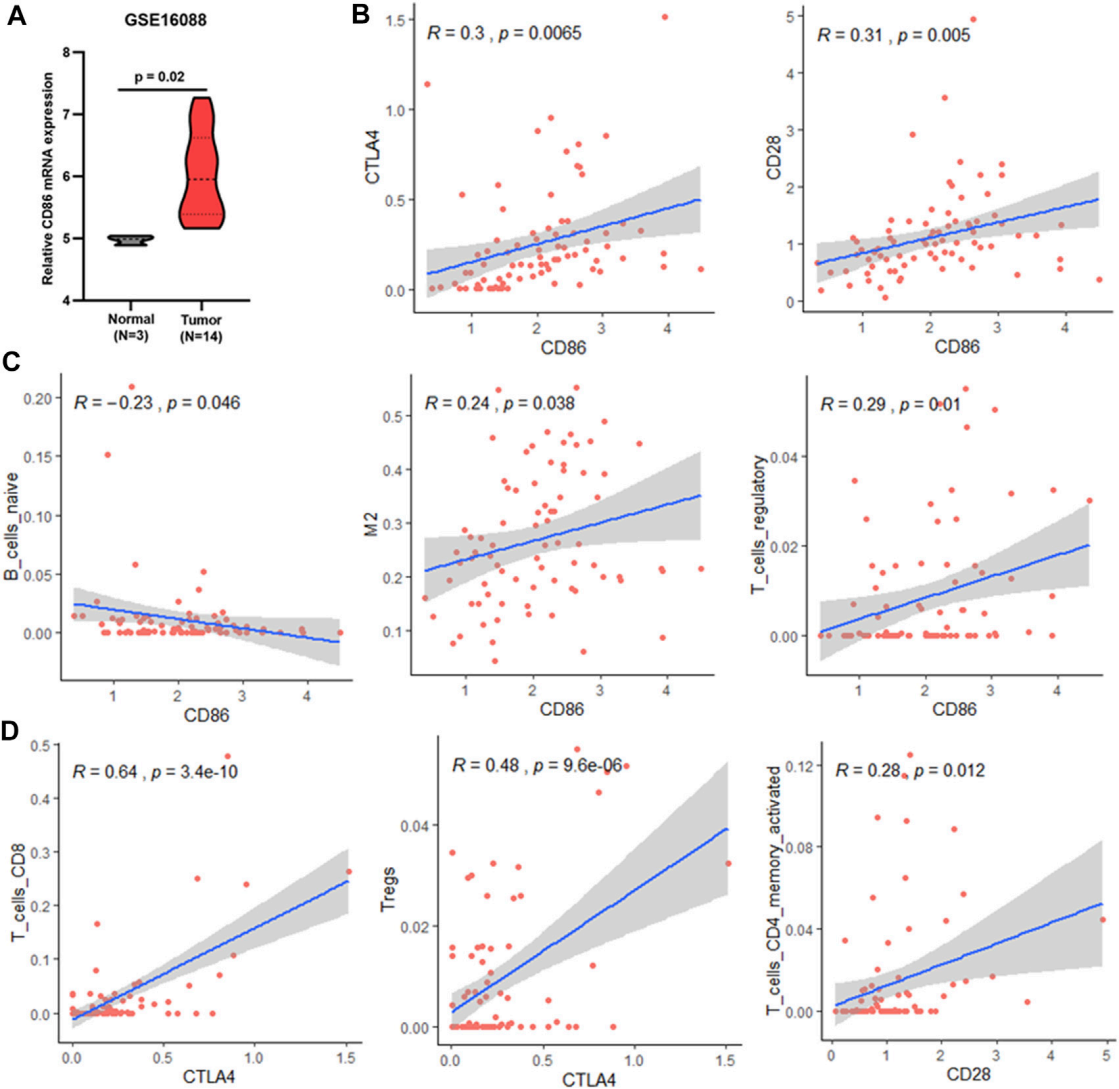
Association between CD86 and TIICs. **(A)** CD86 expression in normal bone and osteosarcoma tissues (GSE16088). **(B)** Correlation between CD86 expression and CTLA4 or CD28 expression in validation cohort (TARGET). **(C)** Associations between CD86 expression and infiltration of naïve B-cells, M2 macrophages, and regulatory T-cells in the validation cohort (TARGET). **(D)** Correlation between CTLA4 or CD28 expression and infiltration of CD8 T-cells, Tregs, and memory CD4 T-cells in the validation cohort (TARGET).

As mentioned above, CD86 was found to be a hub gene and a prognosis-related gene. Our results showed that CD86 mRNA expression in tumor tissues was higher than that in normal bone tissues (Figure 7A), and elevated CD86 expression was beneficial to the prognosis of osteosarcoma (Supplementary Image S1). As a co-stimulatory molecule, CD86 binds to CTLA4 or CD28 receptors and produces coinhibitory or costimulatory signals, respectively; costimulatory signals are necessary for T-cell activation and survival. In this study, CD86 expression was significantly correlated with CTLA4 and CD28 expression in osteosarcoma (Figure 7B). CTLA4 binds more strongly to CD86 than CD28; however, CD86 has a relative preference for CD28 (Pentcheva-Hoang et al., 2004; Esensten et al., 2016). Combining these results, we deduced that the CD86/CD28 stimulatory pathway was dominant in osteosarcoma patients with a good prognosis.

CD86 is expressed in antigen-presenting cells. In this study, CD86 expression was significantly associated with naïve B cells, M2 macrophages, and Tregs (Figure 7C). In osteosarcoma tissues, M0 and M2 macrophages are the major constituents of TIICs (Supplementary Image S2). Therefore, CD86 may be mainly expressed in M2 macrophages in osteosarcoma. The association between CD86 and Tregs may be due to CTLA4 expression on CD4 T cells, CD8 T cells, and Tregs (Rowshanravan et al., 2018). Similar results were observed in this study; CTLA4 expression was significantly associated with CD8 T-cells and Tregs (Figure 7D). The CTLA4 co-inhibitory signal directly prevents T-cell activation and inhibits the activity of other cells through Tregs (Rowshanravan et al., 2018). Therefore, the CTLA4 co-inhibitory signal mainly inhibits the activity of CD8 T-cells in osteosarcoma. Conversely, blocking CTLA4 may benefit the activity of CD8^+^ T-cells and improve prognosis. CD86/CD28 costimulatory signals are necessary for T-cell activation and survival (Esensten et al., 2016). In osteosarcoma, CD28 expression is significantly associated with activated memory CD4 T-cells (Figure 7D). It is possible that the CD86/CD28 costimulatory signal primarily affected the survival and activation of memory CD4 T-cells, which were associated with OS and RFS (Figure 6) and could kill tumor cells (Golubovskaya and Wu, 2016). In summary, blocking CD86/CTLA4 signaling and promoting CD86/CD28 signaling are potential strategies for osteosarcoma immunotherapy.

### Knockdown of PGF Inhibited the Proliferation and Viability of Osteosarcoma Cells

**FIGURE 8:**
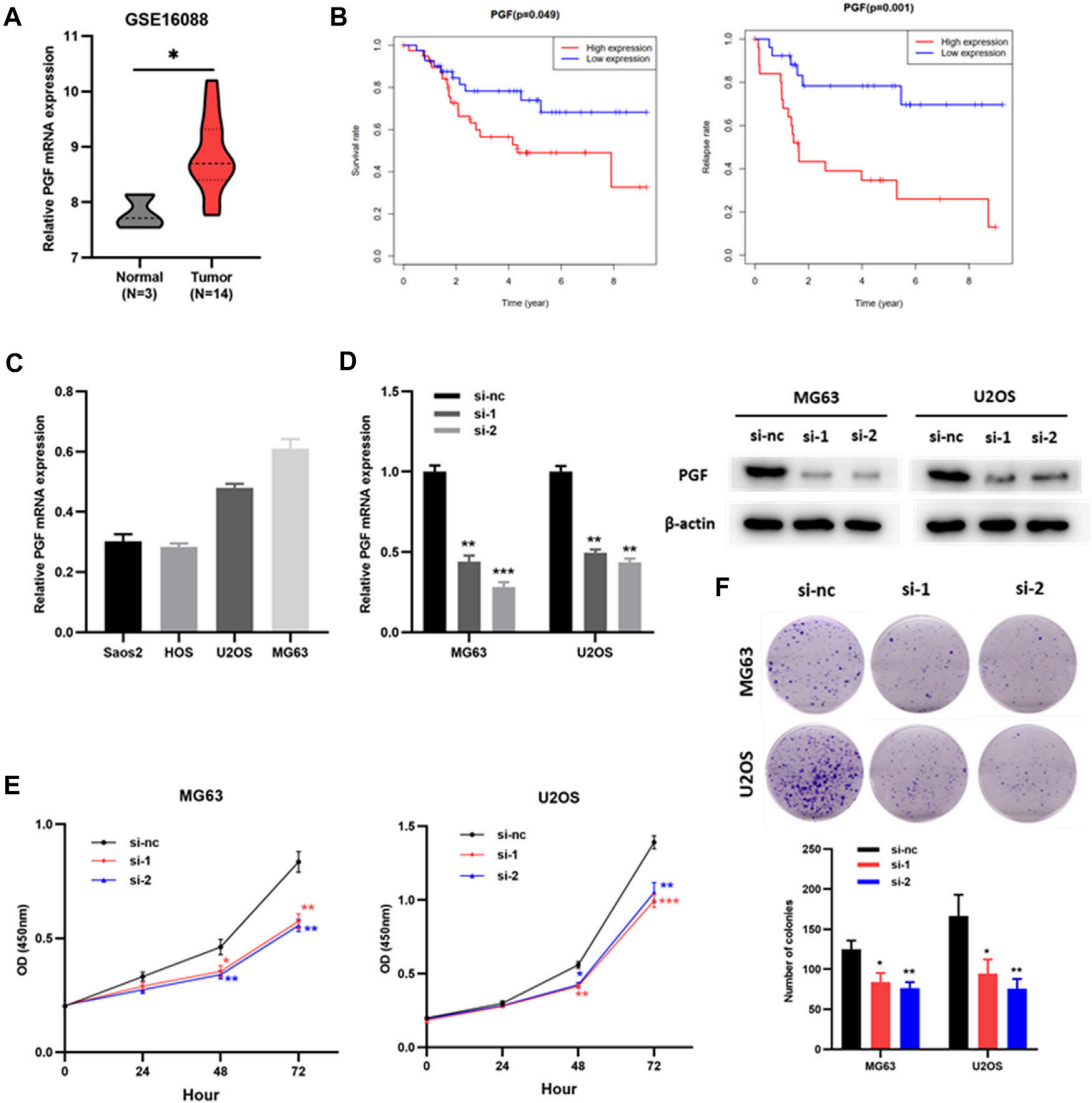
Knockdown of PGF inhibited the proliferation and viability of osteosarcoma cells. **(A)** PGF expression in normal bone tissues and osteosarcoma tissues (GSE16088). **(B)** The OS and RFS analysis of PGF expression in validation cohort (TARGET). **(C)** PGF mRNA expression in osteosarcoma cells. **(D)** PGF knockdown MG63 and U2OS cells were constructed and confirmed by RT-PCR and Western blotting. **(E)** The proliferation ability of MG63 and U2OS cells with PGF (si-nc, si-1 or si-2) *via* CCK8 assays. **(F)** Plate clone formation assays of MG63 and U2OS cells with PGF (si-nc, si-1 or si-2). *p < 0.05, **p < 0.01, ***p < 0.001.

Placental growth factor (PGF) has been identified as an OS- and RFS-related IRG, but its role in osteosarcoma is still unclear. Therefore, we verified the effect of PGF on osteosarcoma cells *in vitro*. PGF mRNA expression was upregulated in tumor tissues compared to that in normal bone tissues (Figure 8A, Supplementary Table S3). Patients with high PGF mRNA levels had poorer OS and RFS than those with low PGF mRNA levels (Figure 8B). Subsequently, PGF mRNA expression in multiple osteosarcoma cell lines was detected by RT-PCR (Figure 8C). MG63 and U2OS cells were used for the subsequent experiments. Next, siRNAs (si-nc, si-1, and si-2) were transfected to MG63 and U2OS cells. RT-PCR and western blotting assays showed that PGF expression was significantly downregulated in osteosarcoma cells (MG63 and U2OS) transfected with si-1 or si-2 compared with those transfected with si-nc (Figure 8D). The results of the CCK8 showed that the proliferation of MG63 and U2OS cells with knockdown PGF (si-1 or si-2) was weaker than that of the control group (si-nc) (Figure 8E). Plate clone formation assays showed that the colony numbers of MG63 and U2OS cells with PGF knockdown were significantly reduced (Figure 8F). This suggested that the knockdown of PGF inhibited the viability of osteosarcoma cells. Taken together, these results show that PGF plays an important role in osteosarcoma progression and may serve as a potential prognostic biomarker.

## Discussion

Immunotherapy has shown excellent therapeutic effects on various malignant tumors, bringing hope to patients; however, to date, immunotherapy for osteosarcoma is still suboptimal (Rodig et al., 2018; Chen et al., 2021). It is urgent to further study the molecular mechanisms of osteosarcoma to identify specific targets and provide new therapeutic directions. Therefore, we performed a comprehensive analysis of IRGs and TIICs associated with osteosarcoma.

First, 129 differentially expressed IRGs were identified from 1793 IRGs; 95 were upregulated and 34 were downregulated. These upregulated and downregulated IRGs may play different roles in osteosarcoma, and GO and KEGG pathway analyses were performed. The results showed that the upregulated IRGs mainly affected antigen processing and presentation and NK cell-mediated cytotoxicity through cytokine and growth factor binding, while the downregulated IRGs mainly regulated receptor signaling pathways through receptor-ligand and growth factor activities. Although these upregulated and downregulated IRGs play different roles in osteosarcoma, they both interact with growth factors. Some growth factors have also been shown to promote osteosarcoma progression (Yang and Zhang, 2013), but core growth factors have yet to be discovered.

Next, eight hub genes were identified by Cytoscape: CXCR4, PDGFRB, CXCL10, B2M, CD86, CSF1R, TYROBP, and FGF2. CXCR4 (Zhu et al., 2018), CSF1R (Smeester et al., 2020), and FGF2 (Yang et al., 2021) have been shown to promote OS progression. PDGFRB (Liu et al., 2018), CXCL10 (Niu et al., 2020), CD86 (Jin et al., 2019), and TYROBP (Wang et al., 2021) were identified as hub genes according to bioinformatic analysis.

To explore the prognostic significance of these differentially expressed IRGs, we performed Cox survival analysis and constructed OS and RFS prediction models for the training cohort (GSE39058). APLNR, TPM2, PGF, CD86, PROCR, and SEMA4D were selected as features for the OS prediction model, and HLA-B and PGF were selected as features for the RFS prediction model. The ROC analysis demonstrated that the OS and RFS prediction models were reliable in selecting patients with osteosarcoma, which was further validated by the TARGET validation cohort. These findings suggest the clinical practicality of OS and RFS prediction models. To enable physicians to predict patients, survival at 1 year, 3 years, and 5 years more intuitively, OS- and RFS-prediction nomograms were constructed.

Previous studies have shown that IRGs are inseparable from TIICs (Zhang and Zhang, 2020). Therefore, we further explored the composition of TIICs in osteosarcoma and the effect of the risk score on TIICs. The results showed that M0 and M2 macrophages were the major constituents of TIICs, and the infiltration proportions of M0 and M2 macrophages in tumor tissues were significantly increased compared with those in normal bone tissues. This also confirmed that macrophages are the main component of immunity in the OS microenvironment (Cersosimo et al., 2020). Survival analysis showed that memory CD4 T-cells were positively correlated with OS and RFS. Therefore, promoting the proliferation and activation of memory CD4 T cells may be a strategy for improving osteosarcoma prognosis. Moreover, the risk score designated by OS- and RFS- prediction models were associated with the infiltration of some TIICs, which suggested that these prognosis-related IRGs influenced immune cell infiltration. However, the key prognosis-related IRGs still need to be explored.

In this study, CD86 was found to be a hub gene and a prognosis-related gene. Importantly, CD86 is an immune checkpoint molecule. Our results showed that CD86 expression was upregulated and associated with a good prognosis. Moreover, CD86 expression depended on its receptors, CTLA4 and CD28, in osteosarcoma. Moreover, in the 22 TIICs, CD28 was only associated with memory CD4 T-cells, which were associated with OS and RFS. In competition, compared with CTLA4, CD86 had a relative preference for CD28 (Esensten et al., 2016). Based on these results, we concluded that CD86/CD28 costimulatory signals played a major role in patients with a good prognosis, possibly by influencing the activation of memory CD4 T-cells. CD86 is expressed in antigen-presenting cells. In this study, CD86 expression was significantly correlated with naïve B cells and M2 macrophages, and M0 and M2 macrophages were the major constituents of TIICs in osteosarcoma. In addition, CD86 expression and the infiltration proportions of M0 and M2 macrophages in tumor tissues were significantly increased. Although dendritic cells (DCs) are common specialized antigen-presenting immune cells (Wylie et al., 2019), the infiltration proportion of DCs was much lower than that of M2 macrophages. Therefore, the upregulation of CD86 expression was mainly due to the increase in M2 infiltration, which may affect the CD86 signaling pathway, CD86/CD28 costimulatory signal, or CD86/CTLA4 co-inhibitory signal. In this study, CTLA4 expression was significantly associated with CD8 T-cells and Tregs, which was consistent with the expression of CTLA4 on CD4, CD8, and Tregs (Rowshanravan et al., 2018). Moreover, the CTLA4 co-inhibitory signal directly prevents T-cell activation and inhibits the activity of other cells through Tregs (Rowshanravan et al., 2018). Therefore, we concluded that CD8 activation by T-cells was directly prevented by the CD86/CTLA4 coinhibitory signal and was indirectly inhibited by Tregs in osteosarcoma. Hence, blocking CTLA4 may benefit the activity of CD8 T-cells and possibly improve prognosis. Thus, blocking CD86/CTLA4 signaling and promoting CD86/CD28 signaling are potential strategies for osteosarcoma immunotherapy.

Moreover, PGF was related to both OS and RFS, but the role of PGF in osteosarcoma remains unclear. The role of PGF in osteosarcoma cells was preliminarily explored. Our results suggest that PGF promotes the proliferation and viability of osteosarcoma cells. Therefore, PGF plays an important role in osteosarcoma progression and may serve as a potential prognostic biomarker. In addition, the depletion of PGF inhibited the transformation from tumor-associated macrophages to M2-like macrophages and remodeled the tumor-immunosuppressive microenvironment to an antitumoral condition in breast cancer (Song et al., 2018). In the present study, PGF expression was positively correlated with the expression of M2 macrophages. Combined with the results of the previous paragraph, we hypothesized that PGF affected CD86 signaling by regulating the polarization of M2 macrophages. More studies are needed to further explore the role and mechanism of PGF and CD86 in osteosarcoma. However, PGF and CD86 signaling deserve our attention.

In conclusion, we comprehensively investigated the prognostic values and potential functions of differentially expressed IRGs in osteosarcoma. OS and RFS prediction models that can reliably predict the prognosis of osteosarcoma patients were developed and validated by the TARGET validation cohort. Seven prognosis-related IRGs and eight hub IRGs were identified. CD86 signaling and PGF may serve as potential specific immune targets in osteosarcoma.

## Data Availability

The original contributions presented in the study are included in the article/Supplementary Material, further inquiries can be directed to the corresponding authors.

## Supplementary Material

Supplementary Image S1 Univariate COX regression analysis of OS and RFS in training cohort (GSE39058).

Supplementary Image S2 The 22 types of TIICs assessed by CIBERSORT algorithm. **(A)** Bar plot of the 22 types of TIICs in GSE16088. **(B)** Bar plot of the 22 types of TIICs in the validation cohort (TARGET).

Supplementary Image S3 Pearson’s correlation heatmap for the seven prognosis-related IRGs and TIICs. Red dots represent a positive correlation, blue dots represent a negative correlation, and the blank represents *p* > 0.05.

Supplementary Table S1 The symbols and categories of 1793 IRGs.

Supplementary Table S2 The sequences of primer and siRNA oligonucleotides.

Supplementary Table S3 Differentially expressed IRGs.
