# Supplementary figures and images for: Development and Validation of Novel Prognostic Models for Immune-Related Genes in Osteosarcoma
Source: Front Mol Biosci. 2022 Apr 6;9:828886. doi: 10.3389/fmolb.2022.828886 (PMC9019688; doi:10.3389/fmolb.2022.828886)

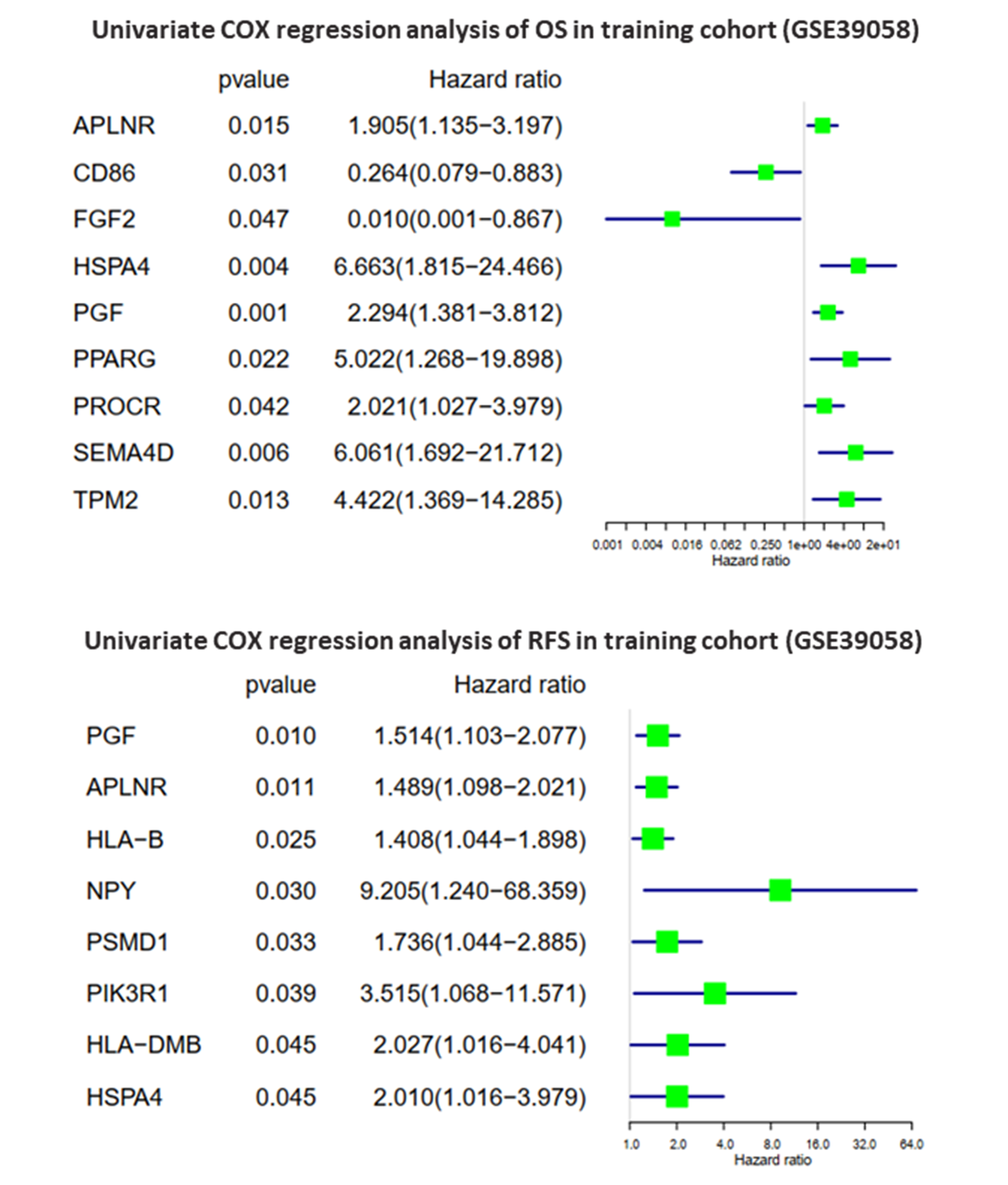

Supplement: Supplementary file 2 [file Image1.TIF]

A

GSE16088

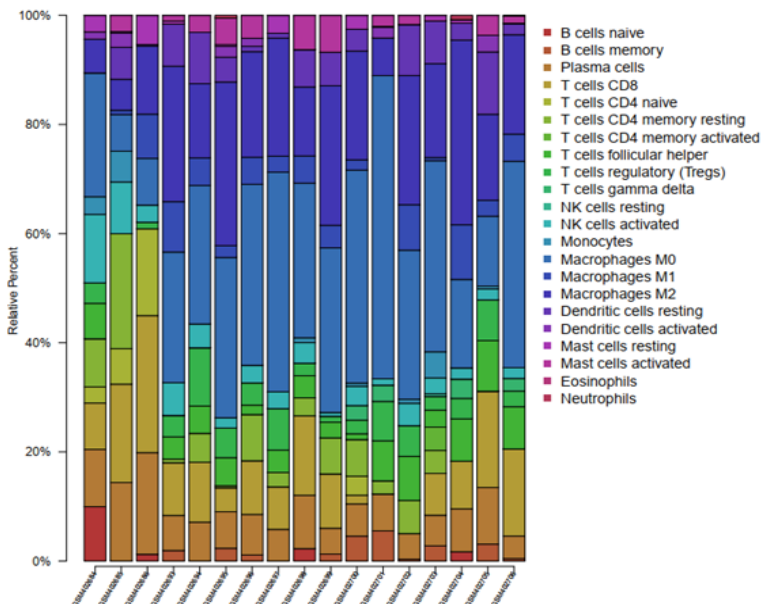

B

TARGET

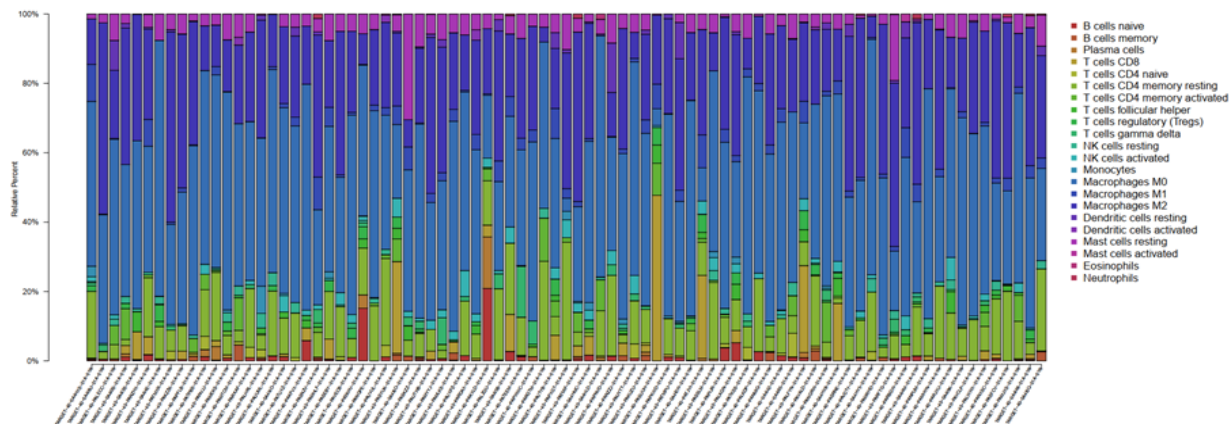

Supplement: Supplementary file 3 [file Image2.PDF]

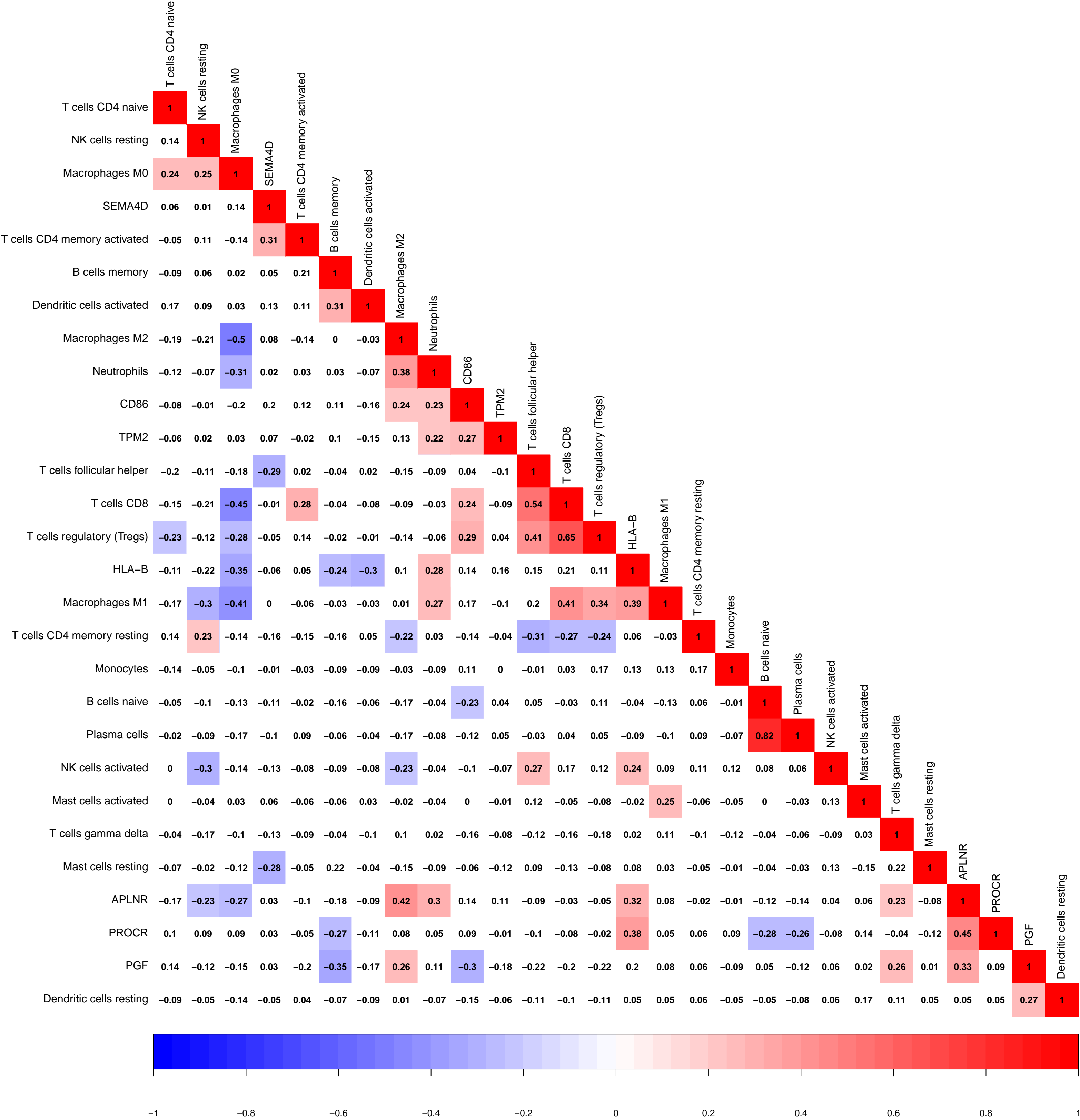

Supplement: Supplementary file 4 [file Image3.PDF]
