# Supplementary material for: Development and Validation of Novel Prognostic Models for Immune-Related Genes in Osteosarcoma
Source: Front Mol Biosci. 2022 Apr 6;9:828886. doi: 10.3389/fmolb.2022.828886 (PMC9019688; doi:10.3389/fmolb.2022.828886)
Supplement: Supplementary file 5 [file Table2.DOCX]

| **Suppl.Table 2.** The sequences of primer and siRNA oligonucleotides | | |
| --- | --- | --- |
| PGF | F | 5′-TGCTGGTCATGAAGCTGTTC-3′ |
|  | R | 5′-GGACACAGGACGGACTGAAT-3′ |
| β-actin | F | 5′-CATGTACGTTGCTATCCAGGC-3′ |
|  | R | 5′-CTCCTTAATGTCACGCACGAT-3′ |
| si-1 |  | GTACCTGCCCTCTATTTAT |
| si-2 |  | GACGTTCTCTCAGCACGTT |
